# Presentation and management of keloid scarring following median sternotomy: a case study

**DOI:** 10.1186/1749-8090-5-122

**Published:** 2010-12-01

**Authors:** Rikesh Patel, Sotiris C Papaspyros, Kalyana C Javangula, Unnikrishnan Nair

**Affiliations:** 1 Leeds General Infirmary, Great George street, Leeds LS13EX, UK

## Abstract

**Introduction:**

Keloid scars following median sternotomy are rare and occur more frequently in pigmented skin. Different management strategies have been described with variable success. We present a case of keloid scar formation following cardiac surgery including our management and the final aesthetic result.

**Case description:**

A 64 year old female of fair complexion underwent mitral valve replacement. The procedure and postoperative recovery were uncomplicated, however, during the following year, thick keloid scars formed over the incision sites. Initial non surgical measures failed to relieve pain and did not offer any tangible aesthetic benefit. Eventually surgical excision was attempted. She presented to our clinic for nine months follow up with significant improvement in pain and aesthetic result.

**Discussion and Evaluation:**

Several theories have attempted to explore the pathophysiology of keloid scar formation. A number of predisposing factors have been documented however none existed in this case. A variety of invasive and non invasive approaches have been described but significant differences in success rates and methodology of investigations still precludes a standardized management protocol.

**Conclusions:**

In this case study a rare presentation of keloid scar has been presented. The variety of methods used to improve pain and aesthetic result demonstrates the propensity of keloid scars to recur and the therapeutic challenges that surgeons have to face in their quest for a satisfactory patient outcome.

## Background

Keloid and hypertrophic scars are benign and fibrotic proliferations which demonstrate abnormal wound-healing responses in susceptible individuals [1]. Scar hypertrophy following midline sternotomy for cardiac surgery is rare, occurring more in pigmented skin. It is estimated that up to 4.5% of the general population suffer from hypertrophic scarring [2]. We describe a patient of fair complexion who developed a thick keloid scar at the sites of sternotomy, chest drains and temporary pacing wire.

## Case presentation

A 64 year old female of Chinese origin underwent uncomplicated mechanical mitral valve replacement in 2002. She was reviewed six weeks following discharge. The sternal wound was healing slowly, but there were no signs of infection.

Past surgical history included sterilisation in 1962, haemorrhoidectomy in 1983 and hysterectomy for fibroids in 1985. None of these operations had resulted in scar hypertrophy. No other significant medical history was present.

She made an uncomplicated recovery from her mitral valve surgery and she was discharged back to her general practitioner (GP). In the next 9 months, she noticed increasing pain and thickness of the sternotomy and previous chest drain scars. She was initially treated by her GP with local anti-inflammatory agents with no noticeable improvement. She searched for other treatment options on the internet and chose to use Cica-Care™ (Smith & Nephew™), a silicone gel sheet for six months with little relief. At that stage her GP referred her to a plastic surgeon and a pain therapist who treated her with local steroid and lidocaine injections for approximately 4 years with no improvement. Eventually she was seen by her cardiologist who referred her back to our cardiothoracic unit.

**Figure 1:**
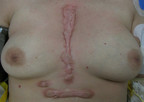
**Keloid scar 4 years post op**. Keloid scarring at site of previous median sternotomy, chest drains and temporary pacing wires.

Her clinical examination confirmed a 12 cm long keloid scar at the site of the sternotomy and two other keloid scars inferiorly at the site of previous chest drains and pacing wires. (Figure 1)

These were extremely tender on light palpation, particularly over the site of the sternal wires. None of the wires were palpable. Following discussion on treatment options she agreed to have surgical excision of the scar and primary closure.

In November 2008, the patient underwent excision of the keloid scar with removal of the sternal wires and reconstruction of the wound with three layers of suture (1 Dexon™ Covidien UK™) under general anaesthetic. Mobilisation of the subcutaneous tissues was required for approximation of the skin edges. Skin was closed with Prolene™ (Ethicon™). Sutures were removed after ten days and a pressure dressing was applied. We were not sure if this patient was allergic to the Monocryl™ and we therefore chose to use a removable material such as Prolene™. The histology of the keloid scar did not show evidence of malignancy.

**Figure 2:**
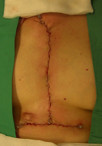
**Immediately post surgical excision**. Surgical excision of scar and sternal wires removal was performed with satisfactory result.

**Figure 3:**
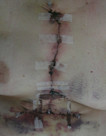
**Day 3 post excision**.

The hypertrophied scar at the pacing wire site was deliberately left untouched in order to compare any future scar formation. (Figure 2) She was discharged home three days later. (Figure 3)

**Figure 4:**
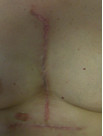
**6 weeks post excision**.

**Figure 5:**
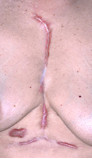
**9 months post excision**. Hypertrophy has recurred however the result is more aesthetically acceptable than the original keloid scar.

The patient was reviewed in the outpatients clinic six weeks later. The wound was healing well with minimal tenderness over the scar sites (Figure 4). Nine months after excision there was evidence of recurrence of her scar, however this was of a lesser degree (Figure 5). The patient felt that pain related to her scar had markedly improved and was satisfied with the overall result.

## Discussion

Several theories exist on the development of hypertrophied or keloid scarring. Predisposing factors include age, sex, race, colour of the skin, anatomic site and post-operative infection. Atiyeh et al. reported keloid and hypertrophic scars as two separate processes that require different therapeutic approaches [3].

The patient in this case report had no apparent predisposition for formation of such pronounced scar hypertrophy. Her skin was fair, the sternotomy wound was not infected, and she had no previous history of scar hypertrophy following surgery.

Elliot et al. studied scar change in presternal areas in fair-skinned population following median sternotomy for open heart surgery [4]. Although they reported no keloid formation, they did observe cases of scar hypertrophy and stretching, which was unrelated to the subcuticular suture material used. They noted scar hypertrophy to occur predominantly over the scar overlying the sternal body and particularly in females. Scar stretching occurred over the lower third of the wound overlying the xiphisternum and upper abdomen.

Methods described for treatment of keloid and hypertrophic scars include local corticosteroid injection, pulsed dye lamp treatment, use of silicone gels and excision of the scar. Currently there is no evidence on superiority of one particular modality [5-8].

In this case report non-invasive management options were initially pursued (silicone elastomer sheeting) due to their relative ease of use and low risk of adverse effects. Although the exact mechanisms of action are unknown, there have been reports of acceptable results [9]. Application of a pressure dressing after surgical excision was used on this patient because simple surgical excision without use of adjuncts is commonly followed by recurrence [10].

Mofikyo et al. were unable to identify a single, reliable and effective protocol regarding management. They reported that surgical excision with post-operative topical steroid injection had low recurrence rates [11]. Sternotomy incisions are relatively immobile and skin closure is free from tension. The usual suture material for skin closure in our unit is monofilament polymer (Monocryl™). There is some evidence that it produces significantly smaller and less reactive scars than other suture materials such as Vicryl-rapide [12].

Durkaya et al. found that the lower half of the wound was more susceptible to scarring regardless of the suture material used, but the upper part was more susceptible to hypertrophy with the use of absorbable sutures [13]. Overall the risk of scar hypertrophy is less with the use of monofilament sutures when compared with absorbable sutures. The relative mobility and increased tension over the xiphoid process was felt to yield a less satisfactory result.

## Conclusion

This case study demonstrates the propensity of keloid scars to recur despite surgical intervention. However there was a significant improvement in aesthetic result and symptoms and this led to patient satisfaction.

## Consent

The authors confirm that written consent has been obtained from patient in order to publish photographs and relevant clinical information included in the submitted manuscript.

## Competing interests

The authors declare that they have no competing interests.

## Authors' contributions

RP is responsible for acquisition of data and writing the original manuscript. SP is responsible for conception and design as well as critical revision of the manuscript, KJ is responsible for conception and design as well as critical revision of the manuscript, UN is responsible for design and conception and critical revision of manuscript. All authors approved the final version submitted.
